# Right Ventricular Myocardial Involvement in Anderson–Fabry Disease at Diagnosis: Evaluation with Three-Dimensional Strain Imaging

**DOI:** 10.3390/life13071571

**Published:** 2023-07-16

**Authors:** Martina Pucci, Velia Iadevaia, Vittoria Gammaldi, Adelaide Iervolino, Luca Maria Capece, Domenico Sciascia, Vittoria Cuomo, Marina Iacono, Daniele Paoletta, Ciro Santoro, Roberta Esposito

**Affiliations:** 1 Department of Clinical Medicine and Surgery, Federico II University Hospital, 80131 Naples, Italy; martina.pucci@unina.it (M.P.);; 2 Department of Advanced Biomedical Sciences, Federico II University Hospital, 80131 Naples, Italy; ciro.santoro@unina.it

**Keywords:** Anderson–Fabry disease, right ventricle, cardiac involvement, three-dimensional speckle-tracking echocardiography (3D-STE)

## Abstract

**Background**: Right ventricular (RV) involvement in Anderson–Fabry disease (AFD) is well known in the advanced stages of the disease RV hypertrophies, but little is known about the early involvement. The aim of our study was to assess RV function in AFD patients at diagnosis. **Methods**: A total of 23 AFD patients and 15 controls comparable for age and sex were recruited. A complete 2D standard echo with 3D volumetric and strain analysis of RV was performed. **Results:** Two patient populations, comparable for clinical baseline characteristics were considered. RV free wall thickness was significantly increased in the AFD group. No significant differences in standard RV indices (TAPSE, transverse diameter, tissue Doppler velocities of the lateral tricuspid annulus) were found. A 3D volumetric analysis showed reduced RV ejection fraction and lower values of longitudinal septal, free wall and global longitudinal strain (GLS) in AFD patients. RV free wall thickness significantly correlated with both free wall RV LS and RV GLS. In multiple linear regression analysis, RV free wall thickness was independently associated with RV GLS even after correction for age and heart rate. **Conclusions:** In AFD patients, 3D echocardiography allows for the identification of early subclinical functional impairment of RV. RV dysfunction is independently associated with RV hypertrophy.

## 1. Introduction

Anderson–Fabry disease (AFD) is a lysosomal storage disorder also known as diffuse angiokeratoma or α-galactosidase A (α-Gal) deficiency. The clinical manifestations are caused by a mutational hit in the GLA gene, located on the X chromosome and encoding for the lysosomal enzyme α-galactosidase A (α-Gal). The mutation causes a progressive intracellular accumulation of globotriaosylceramide (Gb3) and related glycosphingolipids, causing tissue accumulation and multi-organ damage affecting cardiovascular, renal, gastrointestinal, cerebrovascular, neurological, auditory, ocular and skin systems [1]. The pathognomonic cardiac involvement of AFD is a progressive hypertrophic cardiomyopathy associated with heart failure and conduction disturbances [2]. The myocyte’s accumulation of glycosphingolipids is macroscopically expressed as left ventricular hypertrophy (LVH) in the early stages and fibrotic replacement later in the disease.

AFD cardiomyopathy can mimic the clinical and structural features of hypertrophic cardiomyopathy (HCM) [3]. LVH is a more common finding in male patients affected by AFD compared to female ones (53% versus 33% in untreated patients) [4]. Furthermore, it occurs at a younger age in males than females (42 vs. 50.1 years) [5]. 

Accurate medical history and complete physical examination should be obtained at the time of diagnosis. The suspicion of AFD has to be raised in patients with LVH in the presence of the following typical signs and symptoms: corneal opacity, angiokeratoma, hypohidrosis, albuminuria and acroparesthesia. Even so, in some rare cases, there could only be cardiac involvement; thus, the diagnosis of AFD is challenging even with advanced imaging techniques. In the last two decades, enzyme replacement therapy (ERT) or chaperone therapy with Migalastat has been demonstrated to be effective in reducing the accumulation of glycosphingolipids in the different tissues, including cardiac myocytes. ERT should be started in the subclinical disease stages before the development of irreversible target organ damage. Accordingly, early diagnosis and treatment of cardiac involvement in patients and affected family members is a crucial aspect of AFD management [6,7,8,9,10,11,12,13,14].

## 2. Role of Echocardiography in Anderson–Fabry Disease

Standard echocardiography is the first-line imaging for the identification of the typical features of AFD cardiomyopathy, both structural and functional changes such as unexplained left ventricular hypertrophy with RV free wall thickening, preserved left ventricular ejection fraction (LVEF) until end-stage of the disease and progressive diastolic dysfunction [15,16]. However, these echocardiographic findings may occur in other cardiomyopathies with low specificity for AFD. 

### 2.1. Left Ventricular Morphology, Systolic and Diastolic Function

Cardiac involvement in AFD patients is characterized by LV geometry changes starting with concentric remodeling commonly progressing to concentric hypertrophy [17]. A peculiar feature is that hypertrophy of LV walls is more homogeneously distributed, unlike asymmetric HCM, and only infrequently it arises as asymmetric septal hypertrophy or eccentric hypertrophy [17]. LV outflow tract obstruction is infrequent at rest but its incidence increases during effort; it is reported in 43% of patients [18]. Regarding systolic function, LV ejection fraction is usually preserved or even supranormal in the early stages of the disease [19]. In the advanced phases of cardiac involvement, LVEF reduction is associated with a worse prognosis [20]. 

In the past, the *binary sign* (a binary appearance of the LV endocardial border) was considered the pathognomonic hallmark to distinguish AFD patients from those with familial HCM [21]. Despite this, more recent studies have recognized that this sign has very low sensitivity and specificity, only correlating with the degree of septal hypertrophy, and thus not adequately discriminating between patients with AFD and patients with other causes of LVH [22,23].

Regarding LV diastolic function, it is compromised early in patients with AFD. In a report published by Palecek et al., 44% of 81 echocardiographic examinations in 35 patients with AFD had a normal LV filling pattern, while 56% showed an impaired LV diastolic function. Of these, only 4% had a restrictive filling pattern, while 60% had a pseudo-normal filling pattern, and 36% impaired relaxation [24]. The diastolic dysfunction in AFD cardiomyopathy is directly proportional to progressive myocardial wall thickening; its severity increases in the advanced stages of the disease [25,26,27] and represents the substrate for the onset of symptoms [28]. Furthermore, the severity of LV diastolic dysfunction is closely related to NYHA class severity, being a direct echocardiographical parameter to correlate with symptomatic heart failure [29]. Compromised diastolic function, appearing in the early stages of the disease, can anticipate LVH. Therefore, the echocardiographic evaluation with tissue Doppler analysis looking for reduced TDI velocities might be one of the first steps in these patients [27,30]. 

In the setting of preserved LVEF, AFD cardiac involvement reveals an association between diastolic dysfunction indices, namely E/e’ ratio and the presence of late gadolinium enhancement (LGE), an important index of myocardial fibrosis recognized by cardiac magnetic resonance (CMR) imaging. In fact, a cut-off of 14.8 for the septal E/e’ ratio has been demonstrated to be the best predictor of the presence of LGE [31].

Impairment of diastolic function leads to the dilatation of the left atrium. Left atrial dilatation and reduced atrial compliance occurs in the early stages of AFD, even before LVH [31,32,33], making an echocardiographic assessment of the left atrium useful when the disease is still silent or rather subclinical. Atrial damage may be the consequence of the abnormal glycosphingolipids intracellular aggregation that has also been described in atrial myocytes [34]. Usually, atrial enlargement appears as mild or moderate, but in the presence of other significant alterations, such as mitral valve disease, myocardial fibrosis and remarkable LVH, it could appear severe [35]. 

Abnormal glycosphingolipids accumulation may commonly also involve the valvular structures [36]. Aortic and mitral valves are the most affected and rapidly deteriorated valves, probably due to increased pressures in the left heart with respect to the right one [37]. Fortunately, valvular disease is usually mild. In fact, when comparing disease occurrence rate and interventional referral, a study performed with a large series of AFD patients demonstrated valve disease being reported in 14.6% of patients (17% in males, 12% in females), with only 0.4% of cases referred to surgical correction [18].

### 2.2. Right Ventricular Morphology, Systolic and Diastolic Function 

**Figure 1:**
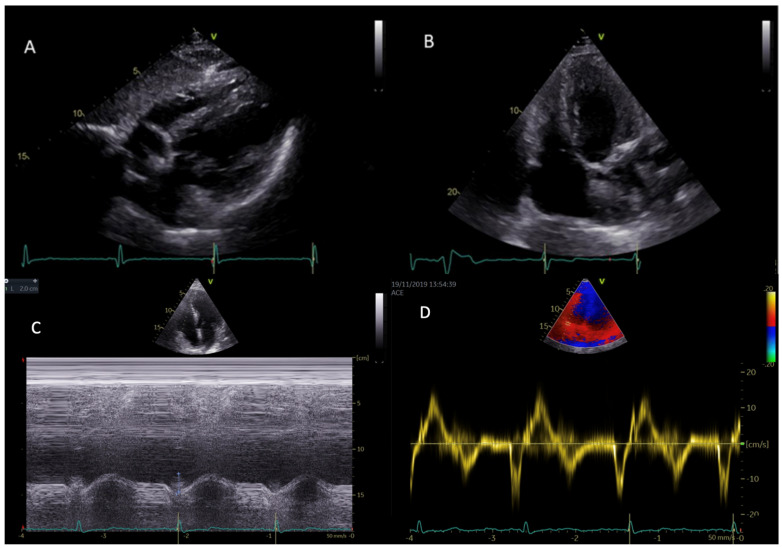
Right and left ventricular hypertrophy in subcostal view (**A**) and in modified apical four-chambers view (**B**) in two different patients with AFD. Parametric indexes of right ventricular function, namely TAPSE (tricuspid annular plane systolic excursion) (**C**) and RVS’ (TDI: tissue Doppler imaging-derived tricuspid lateral annular systolic velocity wave) (**D**) in apical four-chambers view.

Right ventricular hypertrophy (RVH) (Figure 1) is common in patients with AFD and in most cases is related to disease severity and clinical worsening of symptoms. There is no sex difference when comparing the prevalence of RVH to LVH (which is more prevalent in males). Prevalence of RVH is estimated to account for 40–70% of all Fabry cardiac phenotypes. Another important epidemiological data is the strict correlation between RVH and age [38]. 

Current studies in the literature report discordant data on the systolic function of the right ventricle in patients with AFD. Kampmann et al. found that severe RV hypertrophy in Fabry disease is accompanied by severely depressed systolic function as indicated by a depressed tricuspid annulus movement and increased ratio PEP (pulmonary pre-ejection period)/ET (pulmonary ejection time) [39]. The finding of right ventricular systolic dysfunction was not confirmed in subsequent studies. Palecek et al. found that RV wall thickening is not accompanied by RV dilatation or impairment of RV systolic function evaluated by TAPSE (tricuspid annular plane systolic excursion) [40]. These data were confirmed by a subsequent study in which Graziani et al. demonstrated that RV function is typically normal despite hypertrophy. Authors evaluated several parameters such as TAPSE, FAC (fractional area change) and RVS’ (TDI: tissue Doppler imaging-derived tricuspid lateral annular systolic velocity wave) [41].

Regarding the RV diastolic function of patients with AFD, data currently present in the literature agree with the finding of diastolic dysfunction in about half of the cases [39,40,41].

Conventional echo parameters such as fractional area change, tricuspid annular plane excursion and tissue Doppler S’ are recommended measures to assess RV systolic function. However, they have major limitations of being angle-dependent, load-dependent and not fully representing RV global function [42]. Three-dimensional echocardiography shows several advantages when manipulated by expert physicians, including providing more accurate information on diastolic and systolic ventricular volumes and ejection fraction, showing a good correlation with those measured using CMR [43]. In the specific case of AFD, 3D echocardiographical imaging is fundamental for the comprehension and deep understanding of the alterations of parameters such as strain, a measure of myocardial deformation.

### 2.3. Speckle Tracking Echocardiography

Speckle tracking analysis in routine echocardiography has deeply increased diagnostic performances by recognizing intrinsic myocardial dysfunction before LVEF reduction in a subclinical stage [44]. In a report published by Shanks et al., strain and strain rate (SR) analysis have shown to be able to identify AFD patients with reduced myocardial function, independently of LVH [45]. Speckle tracking echocardiography (STE)-derived LV involvement in AFD patients results to be heterogeneous. Our study group [46,47] identified four distinct patterns of longitudinal strain (LS) impairment with reference to regional alterations: (I) normal or near-normal regional LS; (II) reduced LS in septal and anterior regions; (III) reduced LS in both septal/anterior and inferolateral regions; and (IV) reduced LS in the inferolateral region. In any case, in all patients, basal segments resulted more compromised compared to the apical segments of the LV [46].

When comparing HCM and AFD entities, the longitudinal strain pattern is notably different between affected patients. Hypertrophic cardiomyopathy presents a reduction of LS in the septum, while in AFD patients at late-stage, lateral and posterior walls are the most affected ones [48].

The diagnostic work-up of Fabry disease is completed by cardiac magnetic resonance (CMR) which accurately provides differential diagnoses of LV hypertrophy. Late gadolinium enhancement (LGE) can be found in the late stages of the disease. Interestingly, echocardiographic parameters such as the reduction of inferolateral region strain have been shown to predict the presence of LGE on CMR [35]. This allows STE to indirectly predict LGE, i.e., myocardial fibrosis. Of course, this is of particular relevance in patients with implanted devices or end-stage renal disease that are contraindications to CMR.

Recent anatomopathological findings demonstrated that structural changes such as the accumulation of globotriaosylceramide affect all cardiac cell types, including myocardial cells of the right ventricle (RV) and left atrium (LA) [49].

There is evidence in a study performing comprehensive myocardial analyses with 2D-STE and cardiac MRI in AFD patients and a large cohort of healthy subjects that patients with Fabry had significantly lower functional myocardial values of RV and LA, even when conventional cardiac parameters were normal [50]. In addition, functional myocardial alterations detected by 2D-STE were significantly associated with worse symptomatic status and strongly linked to increased cardiac wall thickness [51]. A further more recent study has shown that RV 2D-STE strain in AFD patients in the pre-hypertrophic phase is preserved, unlike AFD patients with overt cardiac involvement. Data from this study showed that peak systolic RV global longitudinal systolic strain and longitudinal peak systolic strain of the RV free wall can be impaired in as much as 41% and 35% of the overall Fabry population and this percentage rise to 58% and 54% when considering patients with Fabry cardiomyopathy [52]. The impairment of RV strain in patients with Fabry cardiomyopathy despite the absence of significant abnormalities of standard measures of RV function highlights the limited sensitivity of the latter parameters to detect initial or minimal signs of RV involvement in Fabry cardiomyopathy.

## 3. Materials and Methods

### 3.1. Study Population

For the purposes of the study, 23 subjects with AFD classical variant were recruited at diagnosis (mean age = 37 ± 14 years, M/F = 15/8) and a group of 15 healthy controls, comparable for age (38 ± 12 years) and sex (M/F = 10/5). All participants gave written informed consent to the procedures before entering the study. The study was approved by the local Ethical Committee (protocol number 1233/19). We excluded patients with relevant cardiac signs/symptoms, patients affected by cardiac risk factors (i.e., diabetes mellitus, dyslipidemia, hypertension), patients already under enzyme replacement therapy and patients with poor echocardiographic imaging. All patients had confirmed diagnosis of AFD: for all subjects, the evaluation of α-GalA activity and the galactosidase alpha (*GLA*) gene test was performed using the dried blood filter paper test. In male patients, diagnosis was made by demonstration of a deficient activity of alfa-galactosidase in plasma; female patients were directly tested for possible mutations in the *GLA* gene, and then evaluated for α-GalA activity when positive at the genetic test.

### 3.2. Echocardiography

Echo Doppler exams were performed by a Vivid E95 ultrasound machine (Horten, Norway) equipped with a 2.5 MHz phased-array transducer according to the American Society of Echocardiography (ASE)/European Association of Cardiovascular Imaging (EACVI) standardization [53]. Blood pressure (BP) and heart rate were measured at the beginning of each echocardiographic exam.

A comprehensive assessment of RV geometry, systolic and diastolic function [54] was performed. RV free wall thickness was measured in 2D linear and 2D-guided M-mode and right ventricular hypertrophy was defined as RV wall thickness >5 mm. RV systolic function was assessed using tricuspid annular plane systolic excursion (TAPSE) by M-mode, tricuspid annular peak systolic velocity (RV S′ wave) by pulsed wave tissue Doppler. A TAPSE <17 mm and RV S′ velocity <9.5 cm/s were considered indicative of RV systolic dysfunction [55].

**Figure 2:**
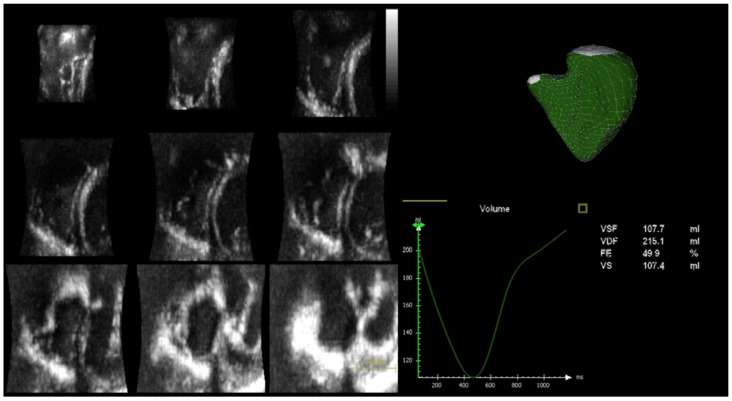
On the left panel, RV full volume 3D 9-slice; on the right panel, determination of end-diastolic and end-systolic volumes (EDV and ESV) and RV ejection fraction (RV EF) by 3D echocardiographic analysis.

A complete 3D analysis (Figure 2) of the right chambers was performed with the determination of end-diastolic and end-systolic volumes (EDV and ESV) and ejection fraction of the RV (RV EF).

### 3.3. Speckle-Tracking Echocardiographic Analysis

The focused RV apical four-chambers view was used for the assessment of peak systolic RV global longitudinal strain (RV GLS), longitudinal peak systolic strain of the RV free wall (RV free wall LS) and longitudinal strain of interventricular septum (RV septal LS) [56]. For STE analysis, images were recorded at ≥50 frames per second to ensure reliable analysis by the software. At an end-systolic frame, a region of interest was traced on the endocardial border by using a point-and-click approach. The adequacy of tracking was verified manually, and the region of interest was adjusted to achieve optimal tracking including the entire myocardial wall and to exclude the pericardium. Then, the software captured the myocardium, automatically tracking its motion and thickening on the subsequent frames.

### 3.4. Statistical Analysis

Statistical analysis was performed by SPSS package, release 12 (SPSS Inc, Chicago, IL, USA). Continuous variables are presented as mean ± SD. Categorical variables are presented as absolute numbers and percentages. Differences in continuous variables between the whole Fabry population vs. controls were analyzed using Student’s *t*-test. Univariate correlates of a given variable were evaluated by least squares linear regression. The null hypothesis was rejected at 2-tailed *p* < 0.05.

## 4. Results

As can be seen from the collection of data in Table 1, the populations under study are overlapping in the characteristics of age, sex, body mass index, systolic blood pressure, diastolic blood pressure, mean blood pressure and heart rate.

As can be seen from data in Table 2, with regard to echocardiographic measurements, we note that functional indices are comparable, such as the basal transverse diameter of the RV, the TAPSE (index measuring the capacity of craniocaudal excursion of the ring of the tricuspid valve during the ventricular systole), RV E/e’ ratio, TDI s’ velocity and TDI e’ velocity of the lateral tricuspid ring.

The data showing the greater discrepancy between the two groups of subjects is certainly the thickness of the free wall of the RV, which significantly increased in the AFD group compared to the 2D echo controls (5.85 ± 1.43 vs. 3.93 ± 0.73, *p* < 0.0001).

As can be seen from the data in Table 3, the three-dimensional volumetric study shows a clear variation in the dimensional parameters of the RV in the AFD group compared to the controls, with higher values of RV EDV (*p* = 0.003) and RV ESV (*p* < 0.0001) with a likely reduced RV EF (*p* = 0.009) in the subjects diseased, as well as the longitudinal septal strain (RV septal LS), free wall strain (RV free wall LS) and the global longitudinal strain (RV GLS) (all with *p* < 0.0001).

As can be seen from data in Table 4, RV free wall thickness correlated significantly with both free wall RV LS (r = 0.35 *p* = 0.05) and RV GLS (r = 0.40, *p* = 0.02).

In the multiple linear regression analysis shown in Table 5, the thickness of the free wall of the RV was independently associated with the RV GLS even after correction for age and heart rate (coefficient β standardized = 0.57, *p* = 0.004) (R.2 cumulative = 0.23, SEE = 4.3%, *p* < 001).

## 5. Discussion

The results of our study clearly show that there is early involvement of the right ventricle in patients with AFD and that the latter can be detected using advanced echocardiographic techniques. In fact, the presence of conflicting data in the literature on the systolic function of the right ventricle in patients with AFD is due to the type of analysis performed: evaluations were carried out using standard echocardiographic parameters such as TAPSE, FAC and RVS. This is the first study that evaluated the right ventricle and its function in AFD patients using 3D echocardiography. This made it possible to overcome the limit linked to the complexity of the geometry of the right ventricle, obtaining a reliable estimate of the end-diastolic, end-systolic volumes and the ejection fraction of the right ventricle. Indeed, several studies have shown that right volumetric evaluation by 3D echocardiography correlates well with the volumes derived from CMR in different groups of patients [57,58,59]. This type of evaluation allowed us to highlight the reduced ejection fraction of RV in patients with AFD at diagnosis compared to the control group.

With regard to strain, several studies have evaluated RV strain [52,60,61]: the most widely used technique to measure RV myocardial strain is currently the 2D strain calculated on the free wall of the right ventricle. However, this assessment has important limitations related to inherent peculiarities of right ventricular geometry, including relatively thinner wall thickness and the need for high-quality RV-focused views for accurate measurement of 2D RV longitudinal strain. In our study, we attempted to overcome these limitations by using 3D strain. The three-dimensional speckle-tracking echocardiography (3D-STE) offers several theoretical advantages as it incorporates the entire right ventricular volume without the need for geometric assumptions, including in the evaluation of the ventricular apex and the right ventricular outflow tract that are not normally visualized in the RV-focused view. This type of analysis allowed us to highlight in patients with AFD at diagnosis a reduction not only in the global longitudinal strain of the right ventricle, but also in the septal longitudinal strain and free wall longitudinal strain compared to the control group.

In our study, a univariate analysis was also performed which evaluated the correlation between the RV free wall thickness and both free wall RV LS and RV GLS; multiple linear regression analysis showed that the thickness of the free wall of the RV was independently associated with the RV GLS even after correction for age and heart rate. This result is extremely important as it underlines how an accurate echocardiographic evaluation using 3D-STE can allow us to highlight, in an early manner, strain alterations that correspond to the degree of hypertrophy of the ventricular walls which, if recognized early, provide the possibility of early treatment.

## 6. Conclusions

Our study allows us, therefore, to conclude that in patients with AFD, 3D echocardiography allows us to identify an early subclinical functional damage, not otherwise detectable by standard ultrasound Doppler.

The strain, and in particular GLS, is able to provide additional information and is therefore crucial to the basic echocardiographic examination, highlighting in an early manner the systolic changes present in conditions, such as hypertrophic cardiomyopathy typical of AFD, and susceptible to a progression, even important and sudden, which obviously wants to be averted with appropriate and timely therapy.

And it is precisely the prospect of an early administration of cardioprotective drugs that is the final objective of this study. In fact, if it is true that it is possible to identify in an early manner a significant reduction of GLS in AFD patients and, therefore, a myocardial compromise indicating the development of imminent cardiomyopathy, it is also true that with the acquisition of such data, it is possible to obtain the appropriate indication to the administration of therapeutic treatment, which prevents the progression of myocardial compromise and safeguards these patients from fatal myocardial damage, typical of the frequent death in AFD.

## Figures and Tables

**Table 1:** Characteristics of the populations under study.

Characteristics	AFD (*n* = 23)	Controls (*n* = 15)	*p*-Value
Gender (M/F)	15/8	10/5	
Age (years)	37.3 ± 14.3	38.5 ± 11.9	0.78
BMI (Kg/m^2^)	26.1 ± 4.3	23.6 ± 2.5	0.05
SBP (mmHg)	126.9 ± 20.9	119.3 ± 9.4	0.20
DBP (mmHg)	77.3 ± 12.1	74.0 ± 9.6	0.37
MBP (mmHg)	93.8 ± 14.4	89.1 ± 8.5	0.25
HR (bpm)	71.4 ± 9.8	73.2 ± 7.6	0.56

(BMI: body mass index, SBP: systolic blood pressure, DBP: diastolic blood pressure, MBP: mean blood pressure, HR: heart rate).

**Table 2:** Standard echocardiographic measurements.

Variables	AFD (*n* = 23)	Controls (*n* = 15)	*p*-Value
RV basal diameter (mm)	36.2 ± 4.6	37.0 ± 5.1	0.44
TAPSE (mm)	23.7 ± 3.7	23.8 ± 3.3	0.93
RV E/e’ ratio	1.45 ± 0.48	1.64 ± 0.25	0.17
TDI s’ vel (cm/s)	14.6 ± 2.3	14.4 ± 1.8	0.73
TDI e’ vel (cm/s)	14.4 ± 3.1	14.3 ± 3.4	0.91
RV wall thickness	5.85 ± 1.43	3.93 ± 0.73	**<0.0001**

(RV: right ventricular; TAPSE: tricuspid annular plane systolic excursion; TDI: tissue Doppler imaging).

**Table 3:** Three-dimensional analysis of the right ventricle.

Variables	AFD (*n* = 18)	Controls (*n* = 14)	*p*-Value
RV EDV (mL)	87.8 ± 27.6	62.8 ± 10.4	**0.003**
RV ESV (mL)	39.4 ± 11.3	24.5 ± 6.7	**<0.0001**
RV SV (L/min)	48.2 ± 19.6	37.8 ± 6.9	0.07
RV EF (%)	54.3 ± 6.5	60.8 ± 6.6	**0.009**
RV Septal LS (%)	17.1 ± 2.9	24.2 ± 5.1	**<0.0001**
RV free wall LS (%)	25.3 ± 3.5	32.3 ± 4.8	**<0.0001**
RV GLS (%)	21.2 ± 2.2	28.2 ± 4.7	**<0.0001**

(RV EDV: right ventricular end-diastolic volume; ESV: end-systolic volume; SV: stroke volume; EF: ejection fraction; LS: longitudinal strain; GLS: global longitudinal strain).

**Table 4:** Univariate relationships of the RV wall thickness in the whole population.

	Variables	R Coefficient	*p*-Value
RV wall thickness	TAPSE	−0.19	0.24
	RV E/a ratio	−0.47	**0.003**
	TDI s’ vel	−0.21	0.19
	TDI e’ vel	−0.24	0.15
	PAPs	0.31	0.06
	3D RV EDV	0.43	**0.01**
	3D RV ESV	0.43	**0.01**
	3D RV EF	−0.21	0.24
	3D RV Septal LS	0.39	0.25
	3D RV Free wall LS	0.35	**0.05**
	3D RV GLS	0.40	**0.02**

(TAPSE: tricuspid annular plane systolic excursion; RV: right ventricular; TDI: tissue Doppler imaging; PAPs: pulmonary artery systolic pressure; EDV: end-diastolic volume; ESV: end-systolic volume; EF: ejection fraction; LS: longitudinal strain; GLS: global longitudinal strain).

**Table 5:** Multiple linear regression analysis.

Dependent Variables	Covariate	β-Coefficient	*p*-Value
RV GLS	Age	−0.39	0.03
	Heart rate	−0.90	0.581
	RV wall thickness	0.57	0.004

(RV: right ventricular; GLS: global longitudinal strain).

## Data Availability

Data presented in this study are available on request due to privacy and ethical restrictions.
